# Supplementary material for: Aluminium in Brain Tissue in Epilepsy: A Case Report from Camelford
Source: Int J Environ Res Public Health. 2019 Jun 16;16(12):2129. doi: 10.3390/ijerph16122129 (PMC6616903; doi:10.3390/ijerph16122129)
Supplement: Supplementary file 1 [file ijerph-16-02129-s001.pdf]

Supplementary

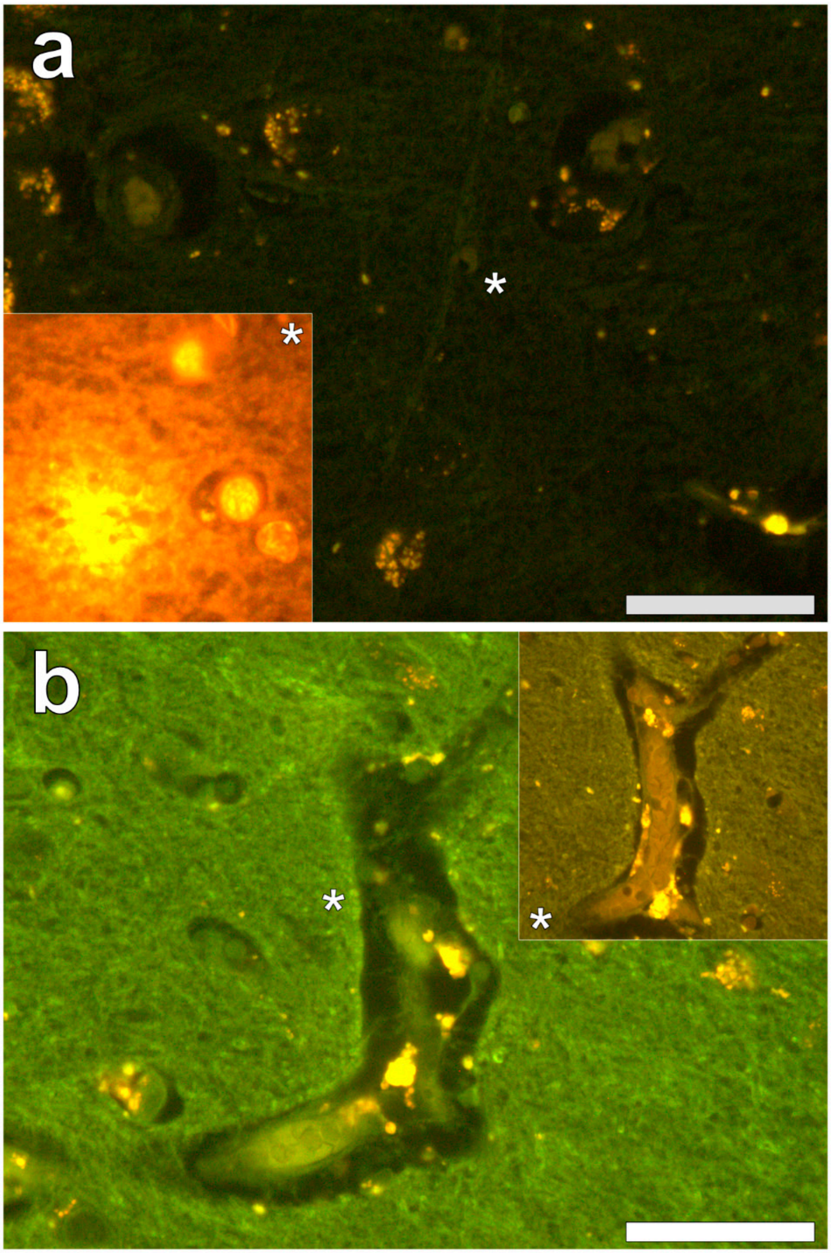

Supplementary Figure 1. Autofluorescence of the frontal and parietal lobe of a 60-year-old male donor diagnosed with epilepsy. A green autofluorescence emission was noted in the frontal and parietal cortex in identical regions, of which the former revealed lumogallion-reactive aluminium in an adjacent 5µm serial section. Lipofuscin was readily apparent producing punctate yellow fluorescence. Inserts depict intracellular aluminium in glial cells and regions found negative for aluminium in lumogallion-stained serial sections (asterisks) of the frontal **(a)** and parietal lobes **(b)**, respectively. Magnification X 400, scale bars: 50µm.

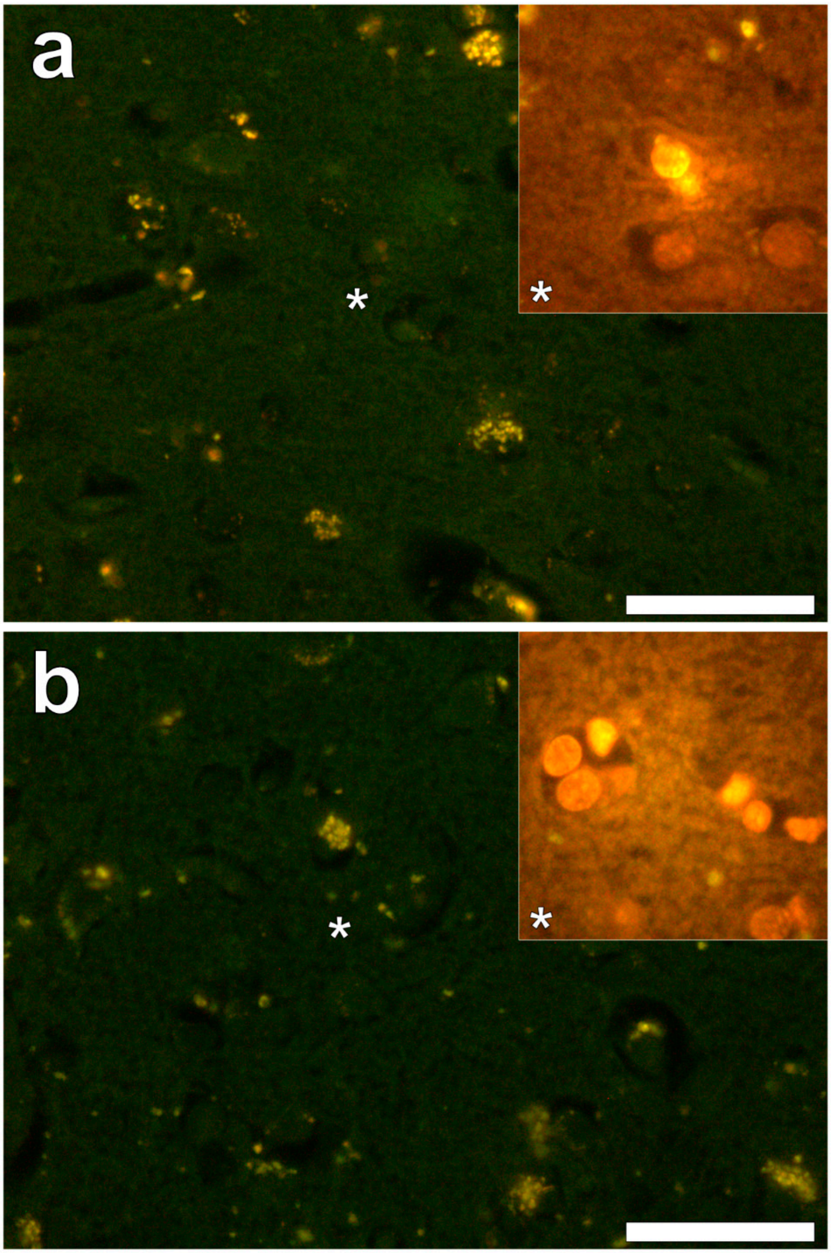

Supplementary Figure 2. Autofluorescence of the occipital cortex of a 60-year-old male donor diagnosed with epilepsy. A weak green autofluorescence emission was noted in the occipital cortex in identical regions that produced intracellular lumogallion-reactive aluminium in glial cells **(a & b)** depicted in adjacent 5μm serial sections as magnified inserts (asterisks). Deposits of lipofuscin were readily apparent producing a weak green/yellow fluorescence emission. Magnification X 400, scale bars: 50μm.

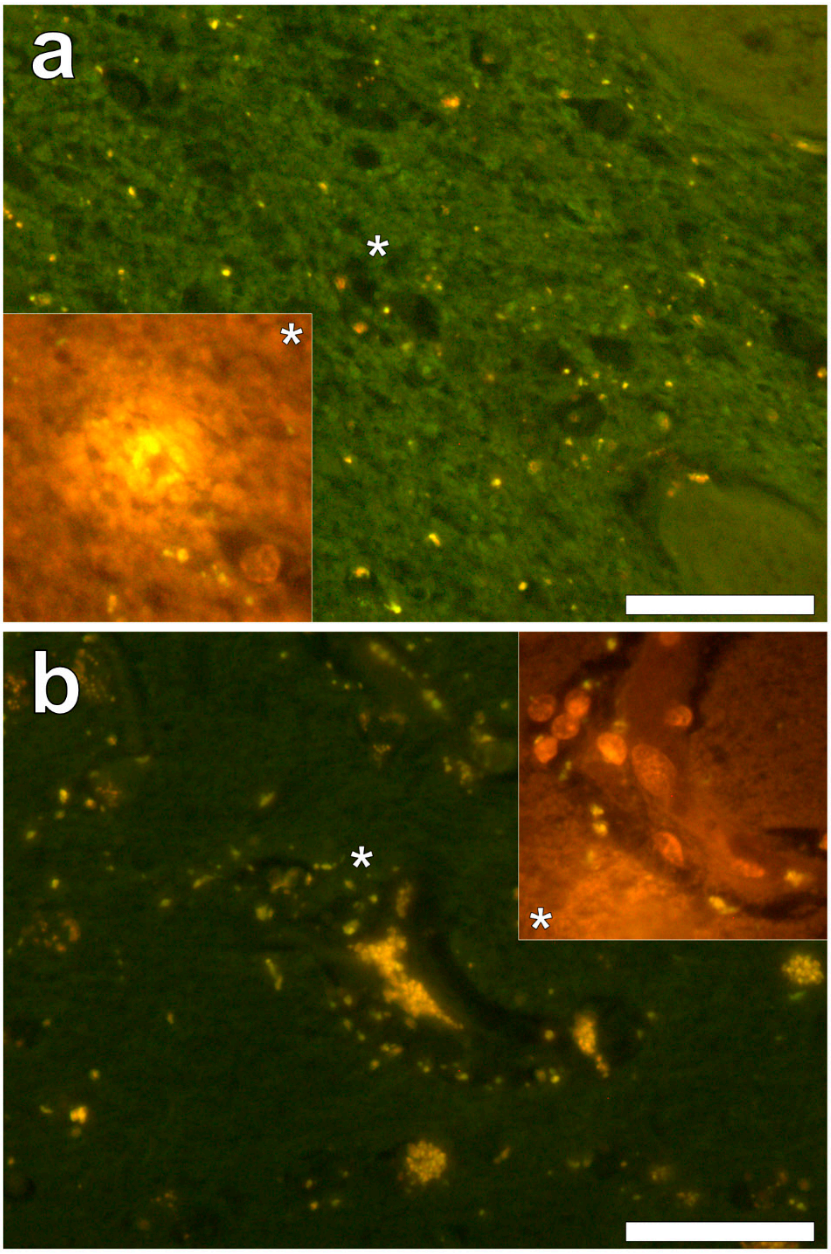

Supplementary Figure 3. Autofluorescence of the temporal lobe of a 60-year-old male donor diagnosed with epilepsy. A green autofluorescence emission of higher intensity was noted in the white matter of the occipital lobe **(a)** versus the cortex **(b)** in identical regions containing extracellular aluminium and intracellular accumulations of the metal ion, as depicted in adjacent 5µm serial sections (asterisks). Deposits of lipofuscin were readily apparent producing a yellow fluorescence emission. Magnification X 400, scale bars: 50µm.

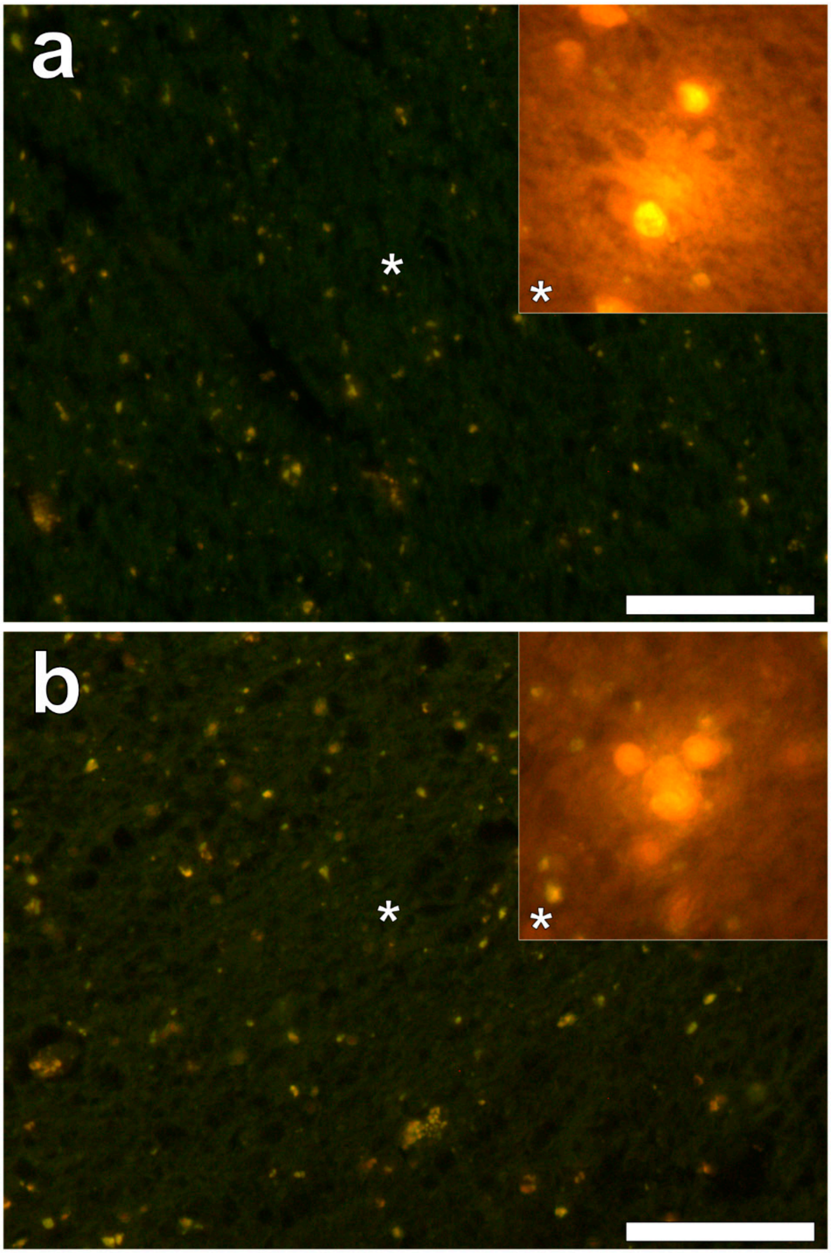

Supplementary Figure 4. Autofluorescence of the hippocampus of a 60-year-old male donor diagnosed with epilepsy. A weak green autofluorescence emission was noted in the occipital cortex, of which lipofuscin was readily revealed via a weak green/yellow fluorescence emission. Magnified inserts depict intracellular lumogallion reactive aluminium within glial cells **(a & b)** in adjacent 5μm serial sections (asterisks), identified upon staining with the fluorophore. Magnification X 400, scale bars: 50μm.
